# Degradation of AZGP1 suppresses apoptosis and facilitates cholangiocarcinoma tumorigenesis via TRIM25

**DOI:** 10.1111/jcmm.18104

**Published:** 2024-01-06

**Authors:** Hyeseon Yun, Hong‐Rae Jeong, Do Yeon Kim, Ji‐Eun You, Ji‐U Lee, Dong‐Hee Kang, Dong‐In Koh, Yea Seong Ryu, SeungGeon Bae, Dong‐Hoon Jin

**Affiliations:** ^1^ Asan Institute for Life Science Asan Medical Center Seoul Korea; ^2^ Department of Pharmacology, AMIST, Asan Medical Center University of Ulsan College of Medicine Seoul Korea; ^3^ Department of Convergence Medicine, Asan Institute for Life Science Asan Medical Center Seoul Korea

**Keywords:** apoptosis, AZGP1, Cholangiocarcinoma, E3 ligase, TRIM25

## Abstract

Alpha‐2‐Glycoprotein 1, Zinc‐binding (AZGP1, ZAG) is a secreted protein that is synthesized by adipocytes and epithelial cells; it is downregulated in several malignancies such as breast, prostate, liver and lung cancers. However, its function remains unclear in cholangiocarcinoma (CCA). Here, we evaluated the impact AZGP1 in CCA using Gene Expression Omnibus (GEO) and GEPIA. In addition, we analysed AZGP1 expression using quantitative reverse transcription PCR and western blotting. Expression of AZGP1 was nearly deficient in CCA patients and cell lines and was associated with poor prognosis. AZGP1 overexpression upregulated apoptosis markers. Co‐immunoprecipitation experiments showed that AZGP1 interacts with tripartite motif‐containing protein 25 (TRIM25), and tissue microarray and bioinformatic analysis showed that AZGP1 is negatively correlated with TRIM25 expression in CCA. Thereafter, TRIM25 knockdown led to AZGP1 upregulation and induced cancer cell apoptosis. TRIM25 targets AZGP1 for degradation by catalysing its ubiquitination. AZGP1 overexpression significantly suppressed tumour growth in a xenograft mouse model. This study findings suggest that AZGP1 is a potential therapeutic target or a diagnostic biomarker for treating patients with CCA.

## 1 INTRODUCTION

Cholangiocarcinoma (CCA) is a malignant tumour occurring in the biliary epithelium^1^ and is anatomically classified as intrahepatic (iCCA), perihilar (pCCA) and distal cholangiocarcinoma (dCCA).^2^ Although the associated risk factors for CCA are cirrhosis, primary sclerosing cholangitis, diabetes, obesity, gallstones and chronic hepatitis B and C, the main cause of CCA remains unclear.^3^, ^4^ Additionally, CCA is obscured by the liver and stomach, therefore confounding its diagnosis via detection or biopsy.^5^ CCA is fatal cancer with a 5‐year overall survival (OS) rate of <5% and its incidence is increasing worldwide.^2^ Although surgical resection is an effective treatment for CCA, approximately 60%–70% of patients are diagnosed at an already advanced stage and are ineligible for surgical resection.^5^, ^6^, ^7^ Gemcitabine and cisplatin are the standard first‐line chemotherapy regimen for the treatment of CCA. However, the prognosis of patients with CCA remains poor.^8^, ^9^ After chemotherapy failure, treatment decisions are driven by several oncogene alterations. Several studies have identified molecular target, such as fibroblast growth factor 2 (*FGFR2*), human epidermal growth factor receptor 2 (*HER2*), metabolic regulators such as isocitrate dehydrogenase 1 and 2 (*IDH1/2*), B–Raf proto‐oncogene (*BRAF*). Pemigatinib, a small molecule inhibitor of *FGFR2*, is the first targeted therapy for CCA approved by the US Food and Drug Administration (FDA). However, pemigatinib has been used in treatments of 10%–15% of CCA patients and the response rate is approximately 30%.^10^, ^11^, ^12^ In the present study, we investigated potential therapeutic targets and biomarkers for the treatment and diagnosis of CCA.

Alpha‐2‐Glycoprotein 1, Zinc‐binding (AZGP1, ZAG) is a 41‐kDa secreted protein, and its encoding gene is located in chromosome 7q22.1.^13^, ^14^ AZGP1 was initially identified through its association with lipid denaturalization in cachexia and obesity.^15^, ^16^ AZGP1 has a similar structure and sequence homology to the extracellular portions of class I major histocompatibility complex (MHC I) heavy chains, suggesting that it plays a role in the immune response.^17^, ^18^ Recently, AZGP1 was reported to be involved in the prognosis and various cancer progressions such as hepatocellular carcinoma (HCC) and pancreatic cancer; it regulates tumour progression through PTEN/Akt signalling and inhibits epithelial‐to‐mesenchymal transition (EMT) through TGFβ1‐ERK2 signalling.^19^, ^20^ Furthermore, microarray and meta‐analyses of clinical relapse and surgical margin‐positive tissue samples show that low AZGP1 expression is associated with poor prognosis.^21^, ^22^ However, no studies have evaluated the value of AZGP1 in CCA.

TRIM25 is a novel RNA‐binding protein and a member of the tripartite motif (TRIM) family of E3 ubiquitin ligase; it regulates proteasomal degradation of PPARγ and is involved in the regulation of adipocyte differentiation.^23^ TRIM25 has a conserved RING domain at the N‐terminus that has E3 ubiquitin ligase activity.^24^, ^25^, ^26^ TRIM25 mediates antiviral signalling through its RNA‐binding activity.^23^, ^24^ TRIM25 promotes cell growth and survival in prostate cancer by regulating p53 signals and in hepatocellular carcinoma by modulating the antioxidant Keap1‐Nrf2 pathway.^27^, ^28^ TRIM25 is an interaction partner of AZGP1.^26^

In this study, AZGP1 expression levels were analysed using the Gene Expression Omnibus (GEO) and the Cancer Genome Atlas (TCGA) databases. We investigated the therapeutic potential of AZGP1 using in CCA cells, xenograft model and explored the mechanism underlying the role of AZGP1 in regulating TRIM25‐induced degradation in CCA.

## 2 MATERIALS AND METHODS

### 2.1 Bigdata analysis

The GEO database (data set GSE26566) from the National Center for Biotechnology Information (NCBI) was used for screening potential candidate genes associated with CCA. The data set contains the expression profiles of 65 normal and 83 tumour sample from CCA. Thresholds for pathway enrichment analysis were *p*‐adjust <0.005 and >∣3∣‐fold change of expression. GEPIA (http://gepia.cancer‐pku.cn/) is an online TCGA database, which was used to analyse AZGP1 and TRIM25 expression and OS in CCA.

### 2.2 Cell culture

Human embryonic kidney HEK293T cells (CVCL_3216), human hepatocellular carcinoma HepG2 cells (CVCL_0027), human CCA KKU‐100 (CVCL_3996), KKU‐213 (CVCL_M261), SNU‐308 (CVCL_5048), SNU‐1079 (CVCL_5008), SNU‐1196 (CVCL_5015), TFK‐1 (CVCL_2214) and TKKK (CVCL_5599) cells were obtained from American Type Culture Collection (ATCC, Manasssa, VA, USA), Korea Cell Line Bank (KCLB, Seoul, Korea) or Japanese Cancer Research Resources Bank (JCRB, Osaka, Japan). The cell lines were maintained in RPMI‐1640 medium or DMEM (WELGENE, Daegu, Korea) supplemented with 10% fetal bovine serum (GIBCO, Grand Island, NY, USA) and 1% penicillin–streptomycin (WELGENE), in a 5% CO_2_ incubator, at 37°C. All cell lines were tested for the presence of mycoplasma using the EZ‐PCR™ Mycoplasma Detection Kit (Biological Industries, #20–700‐20, Israel). Cell line authentication was performed via short tandem repeat (STR) profiling by KCLB.

### 2.3 Quantitative reverse transcription polymerase chain reaction (RT‐PCR)

Total RNA was extracted with TRIzol reagent (Invitrogen, Carlsbad, CA, USA), and 2 μg RNA reverse‐transcribed using the AccuPower® RT Premix (Bioneer, Daejeon, Korea) in a Takara thermal cycler (Takara, China). The primer sequences can be seen in Table S1.

### 2.4 Western blot analysis

Cells were lysed in RIPA buffer on ice. Total protein was electrophoresed by 8%–15% sodium dodecyl sulfate‐polyacrylamide gel electrophoresis (SDS‐PAGE) and transferred to polyvinylidene difluoride (PVDF) membranes for incubation with primary antibodies. Then, the membranes were incubated with HRP‐conjugated secondary antibodies and protein bands detected were using a chemiluminescence western blotting detection kit (GE Healthcare Bio‐Sciences, Sweden). The primary antibodies used in this study were as follows: AZGP1 (Abcam, ab180574, 1:2000), TRIM25 (Proteintech, 67,314‐1‐Ig, 1:5000), β‐Actin (Santa Cruz, SC‐47778, 1:3000), PARP (Cell Signaling Technology, 9542 s, 1:1000), cleaved caspase‐3 (Cell Signaling Technology, 9664 s, 1:1000), cleaved caspase‐9 (Cell Signaling Technology, 9501 s, 1:1000) and HA (Cell Signaling Technology, 3724 s, 1:1000).

### 2.5 Plasmids, shRNA and cell transfection

Empty (Control) and AZGP1 coding regions were cloned into the pCMV‐SPORT6 vector acquired from Addgene. The *AZGP1* point mutations *K84R* and *K91R* were performed using the Muta‐Direct™ Site‐Directed Mutagenesis kit (iNtRON Biotechnology, Seongnam, Korea) and confirmed by DNA sequencing. TRIM25 and its deletion mutations were kindly provided by Dr. V. Narry Kim. Cells were seeded at a density of 3 × 10^5^ cells/60 mm culture dishes and transfected with pCMV‐SPORT6‐Empty(control) or pCMV‐SPORT6‐AZGP1 using Lipofectamine 2000 reagent (Invitrogen, #11668‐019) according to the standard protocol for 24–72 h. Tet‐on inducible expression cell lines were generated using the pcDNA6.0/TR vector (Invitrogen) in KKU‐213 cells. After cells were treated with 20 μg/mL blasticidin (Invivogen, San Diego, CA, USA) for 3 weeks, and TetR stable cell lines were confirmed by western blotting. AZGP1 coding regions were cloned into the pcDNA4/TO vector (Invitrogen), and pcDNA4/TO‐AZGP1 was transfected in TetR overexpressing clones. Transfected cells were selected by treatment with 400 μg/mL zeocin (Invitrogen) for 3 weeks. AZGP1 expression in the selected, stably transfected clones was induced by treating the cells with 1 μg/mL doxycycline (Sigma), and AZGP1 upregulation was confirmed using western blotting.

The small‐interfering RNA (siRNA) negative control(siCtrl) (Bioneer Inc., AccuTarget Negative Control siRNA, SN‐1001) and the short hairpin RNA (shRNA) sequences targeting TRIM25 (shTRIM25) were constructed by Genolution (Seoul, Korea). The shRNA was used as follows: shTRIM25, 5′‐GGUGGAGCAGCUACAACAA‐3′. Cells at 30% confluence in 60 mm culture dishes were transfected with siCtrl or shTRIM25 using Lipofectamine RNAiMAX reagent (Invitrogen, #13778150) according to the standard protocol for 72 h.

### 2.6 Analysis of cell death

Following transfection, cultured cells were prepared as single‐cell suspensions by trypsin. Cells were dissociated by pipetting and mixed with an equal amount of 0.4% trypan blue (Gibco BRL). Stained cells were then counted under a light microscope (Olympus, Tokyo, Japan). Assays were repeated at least three times.

### 2.7 Apoptosis and cell cycle analysis

Cell apoptosis and cell cycle progression were analysed by flow cytometry. After transfection, cells were collected and analysed for apoptosis using a fluorescein isothiocyanate (FITC) Annexin V Apoptosis Detection Kit Ι (BD Biosciences, #BD556547, NJ, USA) according to the manufacturer's instructions. For cell cycle analysis, cells were fixed in 100% methanol at −20°C, washed with PBS and incubated with propidium iodide (50 μg/mL) in PBS containing RNase (100 μg/mL). Stained cells were detected by flow cytometry (Canto II, BD Biosciences), and data analysed using FlowJo software (BD Biosciences).

### 2.8 TUNEL assays

AZGP1 overexpressing KKU‐213 and SNU‐1079 cell lines were seeded into a confocal dish at a density of 1.5 × 10^5^ cells per dish (SPL, Korea). The TUNEL assay was performed using the TUNEL in situ apoptosis kit (Elabscience, #E‐CK‐A320, Houston, TX) according to the manufacturer's instructions. Cells were visualized with a Zeiss LSM 880 confocal microscope.

### 2.9 Immunoprecipitation

Cells were transfected using pSG5‐HA‐TRIM25 wild type and depletion of TRIM25 domain. Cell lysates were incubated with AZGP1 and HA antibodies at 4°C overnight in a rotator. Then, the beads were incubated with protein A/G agarose (Thermo–Fisher Scientific), at 4°C for 4 h, washed in RIPA buffer five times, boiled and analysed by immunoblotting.

### 2.10 Ubiquitination assays

HEK293T cells were grown in 100 mm culture dishes and co‐transfected with/without pSG5‐HA‐ubiquitin, HA‐TRIM25, wild‐type AZGP1 and mutant AZGP1. At 24 h after transfection, the cells were treated with 20 μM MG132 (proteasome inhibitor) for 3 h and harvested. Cell pellets were resuspended in RIPA buffer, and 500 μg of cell lysates was incubated with anti‐AZGP1. After this step, the precipitated pellets were washed with buffer (0.5% Nonidet P‐40, 20 mM Tris–HCl pH 8.0, 120 mM NaCl, 1 mM EDTA), resuspended in 2× SDS sample buffer, resolved by 6% SDS‐PAGE and analysed by western blotting with anti‐HA antibody.

### 2.11 Xenograft models

Female Balbc/nude aged 5–6 weeks (Jabio, Seoul, Korea) were subcutaneously injected into right flank with 5 × 10^6^ cells of doxycycline‐induced AZGP1 expression cells. Cells were injected into groups of three mice. Body weights and tumours were measured twice a week with calliper, respectively. Tumour volumes were calculated using the formula (length×width^2^)/2 = volume (mm^3^). Mice were sacrificed if termination criteria had been reached. All experiments were performed following institutional guidelines established by the Institutional Animal Care and Use Committee at ASAN Laboratory of Animal Research.

### 2.12 Tissue microarrays and Immunohistochemistry

Tissue microarrays were purchased from US Biomax (Rockville, MD, USA). Formalin‐fixed, paraffin‐embedded tissue samples were sectioned into 5 μm slices, which were deparaffinized in xylene, rehydrated and washed with 0.1% TBST, and antigens were retrieved by boiling in target retrieval solution (Dako, CA, USA) for 20 min. Endogenous peroxidase activity was blocked using Dako Cytomatin Peroxidase Blocking Reagent (Dako) for 15 min, and tissue sections were maintained in Serum‐Free Protein Block (Dako) at room temperature for 1 h. After washing, the slides were treated with diaminobenzidine (Dako), followed by counterstaining with Mayer's haematoxylin (Abcam, UK), mounting with histomount and scanning at ×20 magnification using a microscope. Tumour sections were incubated with the following primary antibodies: AZGP1 (Abcam, ab180574, 1:200), cleaved caspase‐3 (Cell Signaling Technology, 9664S, 1:1000), and Ki67 (Abcam, ab16667, 1:200). Positively stained areas were classified as follows: 0, <5%; 1, 5%–25%; 2, 26%–50%; 3, 51%–75%; and 4, >75%. Staining intensity was categorized as follows: 0, negative; 1, weak; 2, moderate; and 3, strong. The score was calculated by multiplying the staining area by the staining intensity, and the results ranged from 0 to 12.

### 2.13 Statistical analysis

Data are presented as the mean ± standard deviation. Two‐sided t‐tests were performed to evaluate *p* values, and *p* < 0.05 was considered statistically significant.

## 3 RESULTS

### 3.1 Downregulation of AZGP1 is associated with poor prognosis

**FIGURE 1:**
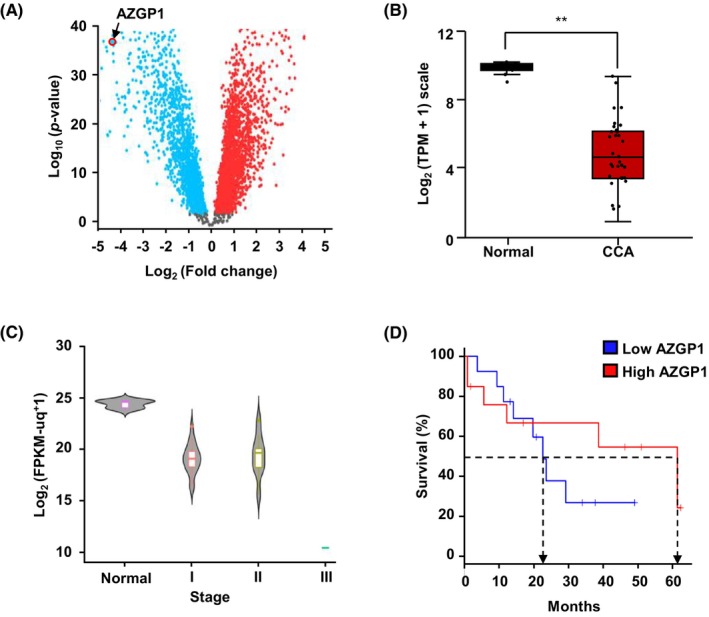
AZGP1 expression is associated with poor prognosis in cholangiocarcinoma (CCA). (A) Alpha‐2‐Glycoprotein 1, Zinc‐Binding (AZGP1) expression was obtained from the Gene Expression Omnibus (GEO) database (GSE26566). (B) Analysis of the GEPIA database showed that AZGP1 expression is downregulated in CCA. (C) The mRNA levels of AZGP1 in various stages of CCA. (D) Kaplan–Meier curve of high and low AZGP1 expression in CCA patient groups (log‐rank *p* = 0.37, *p*(HR) = 0.39, compared with normal controls). **p < 0.01 indicates significant differences from the control group.

In previous reports, microarray analysis of patients with CCA identified that AZGP1 is one of downregulated gene.^29^ Its function in CCA is unclear. Transcriptome analysis of 65 non‐cancerous and 83 CCA samples using the GEO database from the NCBI, and construction of a volcano plot showed that AZGP1 is downregulated in CCA (Figure 1A). Further analysis GEPIA from TCGA^30^ confirmed the GEO database results, showing that AZGP1 is downregulated in CCA compared with normal samples (Figure 1B). In addition, AZGP1 expression decreased in relation to increasing tumour stage of development (Figure 1C), and low expression of AZGP1 was correlated with poor OS in patients with CCA (Figure 1D). Taken together, these results indicate that AZGP1 is downregulated in CCA and associated with poor OS.

### 3.2 Overexpression of AZGP1‐induced apoptotic cell death in CCA

**FIGURE 2:**
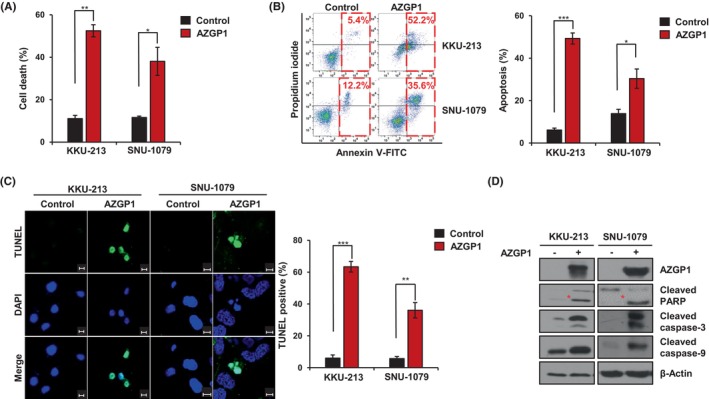
AZGP1 Induces apoptotic cell death in CCA. (A) KKU‐213 and SNU‐1079 cells were transfected with AZGP1, and cell death was determined by hemocytometry. (B) Analysis of apoptosis by Annexin‐V/PI staining and flow cytometry; bar charts were drawn to quantify three separate flow cytometry experiments. (C) TUNEL assay of apoptotic KKU‐213 and SNU‐1079 cells and quantification of three independent experiments using bar charts. Scale bar: 10 μm. (D) Western blot analysis of AZGP1, cleaved PARP, cleaved caspase‐3, cleaved caspase‐9 and β‐Actin in KKU‐213 and SNU‐1079 cells with or without AZGP1 expression. *p < 0.05, **p < 0.01, ***p < 0.005 indicate significant differences from the control group.

The functional role of AZGP1 was examined by overexpressing the protein in CCA cell lines. We found that cell death was higher in cells overexpressing AZGP1 than in the controls (Figure 2A). We confirmed that cell death represents apoptosis through Annexin‐V/PI staining and the TUNEL assay (Figure 2B,C). The rate of apoptosis was 5.4% and 12.2% in control KKU‐213 and SNU‐1079 cells, respectively, and it increased to 52.2% and 35.6% in AZGP1 overexpressing cells (Figure 2B). Moreover, the number of TUNEL positive cells was higher in AZGP1 overexpresseing cells than in the control (Figure 2C). Immunoblotting data revealed that AZGP1 overexpression activated the cleavage of PARP, caspase‐3 and caspase‐9 in KKU‐213 and SNU‐1079 cells (Figure 2D). To identify the cell death mechanism in AZGP1 overexpressing cells, cell cycle progression was analysed by PI staining and flow cytometry. The proportion of cells in sub‐G1 phase was less than 2% in control cells, whereas it increased in AZGP1 overexpressing cells (Figure S1A). AZGP1 overexpression decreased the cell proliferation rate in a dose‐dependent manner (Figure S1B). These results indicate that AZGP1 overexpression activates apoptosis and suppresses cell proliferation in CCA cells.

### 3.3 AZGP1 expression is negatively correlated with TRIM25 in CCA

**FIGURE 3:**
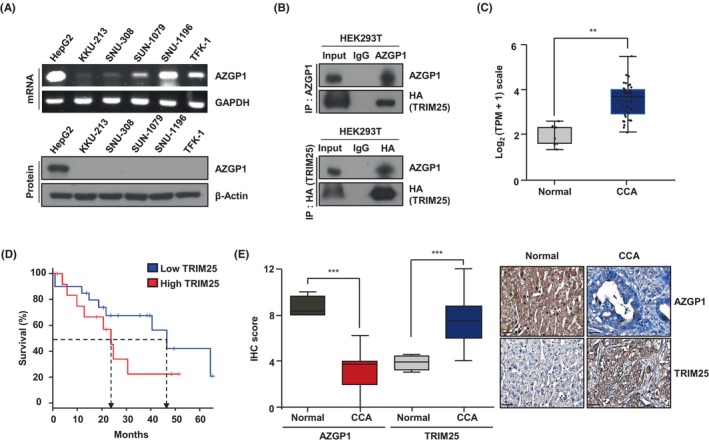
AZGP1 and TRIM25 are associated with immunoprecipitation (IP) and immunohistochemistry (IHC). (A) AZGP1 mRNA and protein expression in CCA cells. (B) Co‐IP assay of interaction between AZGP1 and TRIM25 in HEK293T cells. (C) Box plot of TRIM25 expression obtained from the GEPIA databases. (D) The GEPIA database showed a Kaplan–Meier curve for TRIM25 high and low expression group of CCA (log‐rank *p* = 0.17, compared to normal controls). (E) IHC analysis of AZGP1 and TRIM25 expression in adjacent normal tissue and CCA tissue. Bar charts show quantitative data from the average of IHC scoring (Normal, *n* = 4; CCA, *n* = 63). ***p < 0.005 indicates significant differences from the normal.

AZGP1 mRNA expression was detected in CCA cell lines, whereas the AZGP1 protein was undetectable except in the positive control HepG2 cells (Figure 3A), suggesting that the AZGP1 protein is degraded in CCA. Considering previous findings showing that activation of AZGP1 involves TRIM25 and a novel RNA‐binding domain,^31^ we hypothesized that TRIM25 may be involved in the degradation of AZGP1. Co‐immunoprecipitation (Co‐IP) experiments confirmed that AZGP1 interacts with TRIM25 (Figure 3B). Analysis of TCGA database through GEPIA indicated that high expression levels of TRIM25 are correlated with poor OS in CCA patients (Figure 3C,D). Immunohistochemistry (IHC) analysis of the expression of AZGP1 and TRIM25 in CCA tissue microarrays showed that AZGP1 staining intensity was high in adjacent normal tissues but was barely detected in CCA tissues, whereas TRIM25 was detected at high levels in CCA tissues (Figure 3E). In contrast to AZGP1 expression, TRIM25 was expressed in CCA at the mRNA and protein levels, as determined by RT‐PCR and western blotting (Figure S2A). TCGA database analysis showed a negative correlation between AZGP1 and TRIM25 in CCA (Figure S2B). these results indicate that the AZGP1 protein is downregulated in CCA and that it interacts with TRIM25, an E3 ligase.

### 3.4 Degradation of AZGP1 is mediated by TRIM25 via its PRY/SPRY domain

**FIGURE 4:**
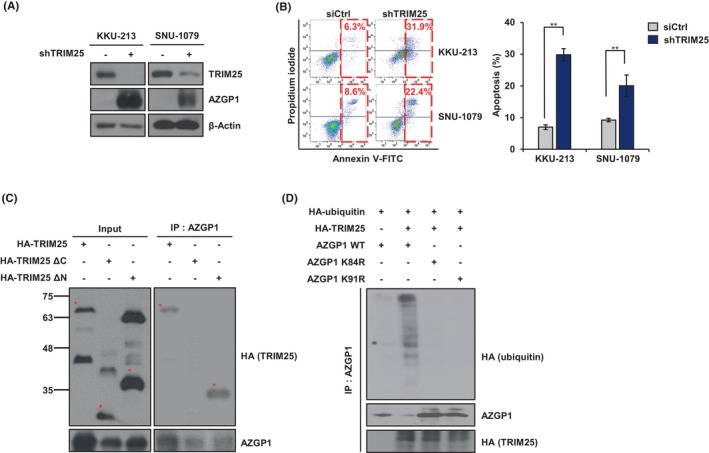
AZGP1 degradation is mediated by TRIM25 PRY/SPRY domain. (A) Western blot analysis of AZGP1, TRIM25 and β‐Actin in KKU‐213 and SNU‐1079 cells with and without TRIM25. (B) Apoptotic cell death analysis of CCA cells with and without TRIM25 knockdown using Annexin‐V/PI staining and flow cytometry, and quantification of three independent flow cytometry experiments by bar charts. (C) HEK293T cells were transfected with TRIM25 or TRIM25 mutants, followed by AZGP1 IP and western blots analysis of AZGP1 and TRIM25‐HA expression. (D) HEK293T cells were transfected with the indicated plasmids. Wild‐type and mutant AZPG1 proteins were analysed by western blotting with indicated antibodies. All experiments were performed 72 h after TRIM25 knockdown in CCA cells. Bar charts show quantitative data from the average of three independent experiments. **p < 0.01 indicates significant differences from the control group.

To clarify the role of TRIM25 in AZGP1 protein degradation, we tested whether TRIM25 could rescue AZGP1 expression. Knockdown of TRIM25 upregulated AZGP1 expression in CCA cells (Figure 4A) and increased apoptotic cell death, as determined by Annexin‐V/PI staining (Figure 4B). To identify which region of TRIM25 is necessary for the interaction with AZGP1, we generated a series of truncated mutants of TRIM25, such as *TRIM25 ΔN* mutant (containing the PRY/SPRY domain), *TRIM25 ΔC* mutant (containing the RING/BB domain)^32^ or TRIM25‐full length fused with HA‐labelled plasmids (Figure S3). These plasmids were co‐transfected with HA‐labelled TRIM25 and TRIM25 mutant into HEK293T cells, Co‐IP experiments showed that only the TRIM25 PRY/SPRY domain interacted with AZGP1 (Figure 4C). This confirmed that TRIM25, and E3 ubiquitin ligase, was involved in the degradation of AZGP1. Additionally, we found AZGP1 degradation sites were identified using protein ubiquitination site prediction tools such as UbPred (http://ubpred.org/). We then constructed AZGP1 mutants in which Lys 84 or Lys 91 was changed to Arg (Figure S3). Immunoprecipitation results showed that TRIM25 catalyses the ubiquitination of AZGP1, resulting in its degradation, as demonstrated by reduced ubiquitination of AZGP1 mutants (Figure 4D). Taken together, these results demonstrate that TRIM25 affects apoptosis by regulating AZGP1 protein levels in CCA, and that AZGP1 binds TRIM25 PRY/SPRY domain, which mediates its ubiquitination and proteasomal degradation.

### 3.5 Overexpression of AZGP1 suppresses tumour growth in vivo

**FIGURE 5:**
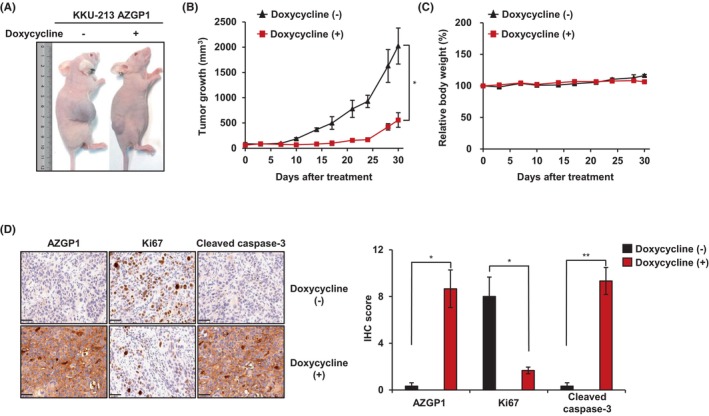
Overexpression of AZGP1 suppresses tumour growth in vivo. (A) Representative images of mice injected with doxycycline‐induced AZGP1 expression cells. (B) The tumour growth curve of the implanted subcutaneous tumours. Tumour growth was measured twice per week. (C) The body weight of mice was measured twice per week. (D) IHC staining for AZGP1, Ki67 and cleaved caspase‐3 in doxycycline‐induced AZGP1 expression cells. Three independent experiments were quantified, and data were expressed in bar charts. Scale bar: 50 μm. *p < 0.05, **p < 0.01 indicate significant differences from the control group.

We hypothesized that upregulation of AZGP1 in vivo would stimulate apoptosis and suppress tumour growth. We established doxycycline‐induced AZGP1 expression cell lines, which were subcutaneously injected into nude mice. When tumours reached 100 mm^3^ in size, mice were randomly assigned to vehicle control or doxycycline‐treatment groups, which received 20 mg/kg doxycycline daily. Tumour growth and size were significantly lower in the doxycycline‐induced AZGP1 expression groups than in the vehicle‐treated groups. Body weight did not differ significantly between vehicle and doxycycline‐treated groups (Figure 5A–C). Additionally, IHC staining showed that the expression of AZGP1 and cleaved caspase‐3 was enhanced while the levels of Ki67 decreased in the doxycycline‐induced AZGP1 expression group (Figure 5D). Collectively, these findings indicated that AZGP1 suppressed CCA tumour growth in vivo.

## 4 DISCUSSION

CCA is a rare malignancy caused by the bile duct epithelium. It is difficult to diagnose owing to its clinical character, the low specificity of modalities and locational characteristics. Through computed tomography (CT) and dynamic magnetic resonance imaging (MRI), CCA is typically detected in its third and fourth stages and largely negates possible curative surgical treatment.^5^, ^33^, ^34^ The first‐line therapy for CCA consists of a combination of cisplatin and gemcitabine chemotherapy, which only slightly slows down tumour progression. Moreover, most patients experience chemotherapy resistance.^2^, ^35^ The frequent failure of chemotherapy prompted research into tumour genomics and the identification of targeted therapies.^36^ Studies aimed at elucidating the molecular mechanism underlying CCA are critical for identifying novel biomarkers and for improving the outcome of this fatal disease Microarray analysis of eight pairs of patients with CCA identified 47 downregulated genes including AZGP1.^29^ However, the biological functions of AZGP1 in CCA remain unclear.

AZGP1 is a soluble and secreted protein classified as an adipokine. It is present in several organs, including adipose tissue, and is involved in fat degeneration.^37^ AZGP1 acts as a tumour suppressor in breast, prostate, gastric, liver and pancreatic cancer. It functions as a tumour suppressor by suppressing migration, invasion and reducing proliferation. Furthermore, AZGP1 inhibits TGF‐β mediated ERK2 phosphorylation, thereby inducing depressing EMT and invasion.^13^, ^20^ Loss of AZGP1 can activate the PTEN/Akt and CD44 pathways, thereby promoting metastasis and migration, and it is associated with poor prognosis.^19^ Furthermore, AZGP1 overexpression suppresses invasion and cell proliferation by inhibiting MMP‐2 and MMP‐9 expression, and it inhibits the activation of mTOR pathway.^38^ Accordingly, loss of AZGP1 promotes EMT, accompanied by increased invasion, decreased apoptosis and proliferation, pro‐survival signals and a shift in energy metabolism.^13^, ^20^ The possibility of AZGP1 as a biomarker in various carcinomas has been suggested.^16^, ^20^, ^22^, ^39^, ^40^

In this study, analysis of the GEO and TCGA datasets demonstrated that downregulation of AZGP1 leads to a poor prognosis in patients with CCA (Figure 1). Similar results were obtained in the analysis of RNA and protein in CCA cells. To evaluate its role in AZGP1, overexpression of AZGP1 in CCA cells induced apoptotic cell death and inhibited cell proliferation (Figures 2 and 3). To determine the mechanism underlying AZGP1 downregulation, we analysed the physical interaction between AZGP1 and TRIM25 in CCA (Figures 3 and 4). TRIM25 is an E3 ligase that is involved in adipocyte differentiation and promotes cancer cell growth. We confirmed that TRIM25 knockdown upregulated AZGP1 and promoted apoptotic cell death and demonstrated that AZGP1 interacts with the TRIM25 PARP‐domain, resulting in its ubiquitination (Figure 4). We generated a xenograft tumour model to demonstrate that AZGP1 suppresses tumour progression (Figure 5). Taken together, these results suggest that AZGP1 initiates apoptotic cell death and sub‐G1 arrest, thereby affecting cell proliferation in CCA, and its activity is inhibited by TRIM25.

In this study, we aimed to overcome the limitations of existing CCA treatments by studying AZGP1 as a novel therapeutic target or diagnostic biomarker in CCA and demonstrated that AZGP1 acts as a tumour suppressor in CCA.

## AUTHOR CONTRIBUTIONS

**Dong‐Hoon Jin:** Conceptualization (equal); supervision (lead); visualization (equal); writing – review and editing (lead). **Hyeseon Yun:** Conceptualization (equal); investigation (equal); methodology (equal); validation (equal); visualization (equal); writing – original draft (equal). **Hong‐Rae Jeong:** Conceptualization (equal); investigation (equal); methodology (equal); validation (equal); visualization (equal); writing – original draft (equal). **Do Yeon Kim:** Conceptualization (equal); visualization (equal). **Ji‐Eun You:** Conceptualization (equal); validation (equal). **JI‐U Lee:** Validation (equal). **Dong‐Hee Kang:** Validation (equal). **Dong‐In Koh:** Conceptualization (equal); investigation (equal); visualization (equal). **Yea Seong Ryu:** Conceptualization (equal); investigation (equal); visualization (equal). **SeungGeon Bae:** Visualization (equal).

## CONFLICT OF INTEREST STATEMENT

The authors declare no competing financial interest.

## Supporting information


Figure S1

Figure S2

Figure S3

Table S1

## Data Availability

All data generated or analysed during this study have been included in this article Additionally, the data that support the findings of this study are available from the corresponding author upon reasonable request.
