# Supplementary figures and images for: Degradation of AZGP1 suppresses apoptosis and facilitates cholangiocarcinoma tumorigenesis via TRIM25
Source: J Cell Mol Med. 2024 Jan 6;28(3):e18104. doi: 10.1111/jcmm.18104 (PMC10844717; doi:10.1111/jcmm.18104)

Supple Fig 1.

(A)

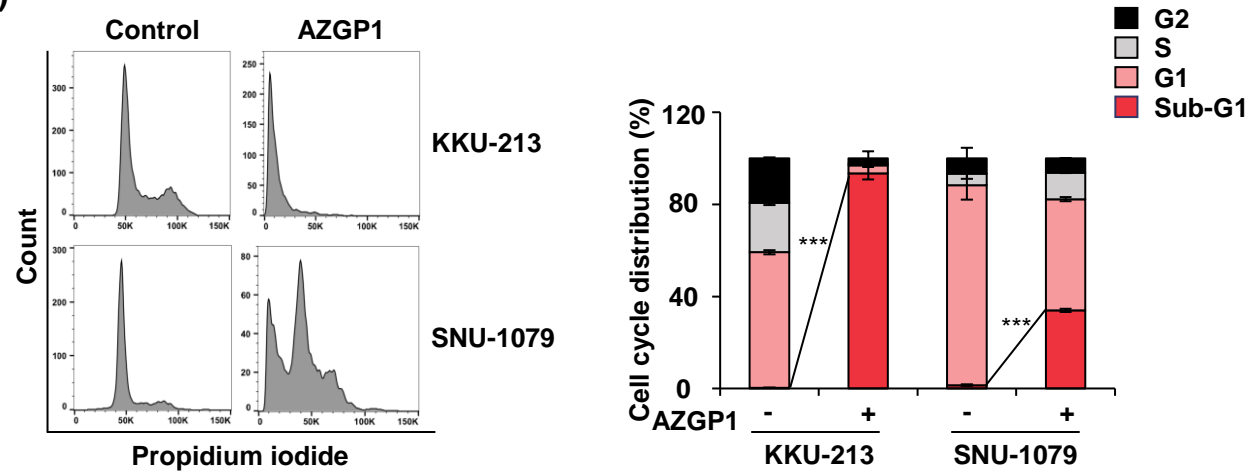

(B)

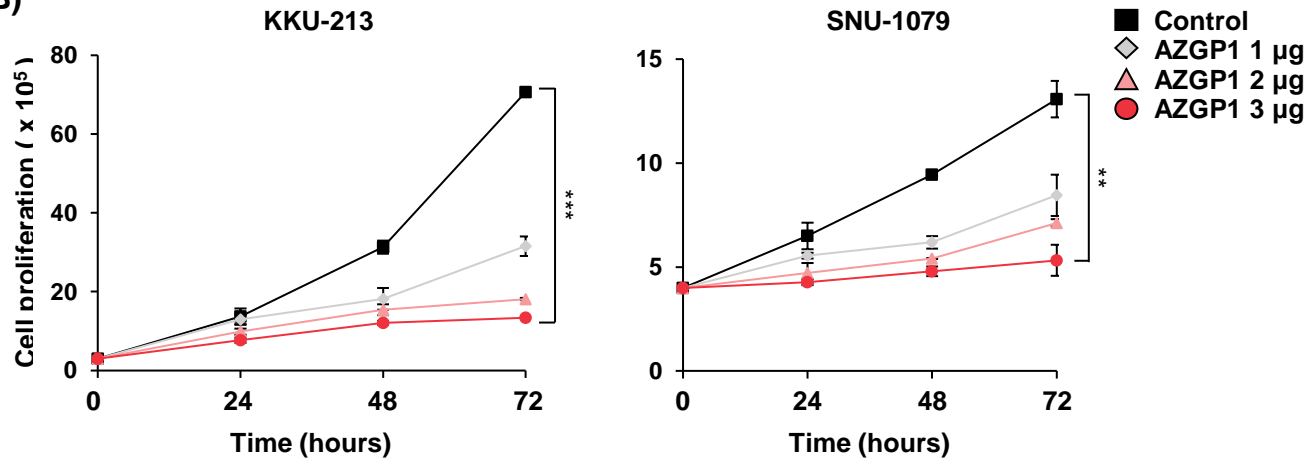

Supplement: Supplementary file 1 — Figure S1 [file JCMM-28-e18104-s004.pdf]

Supple Fig 2.

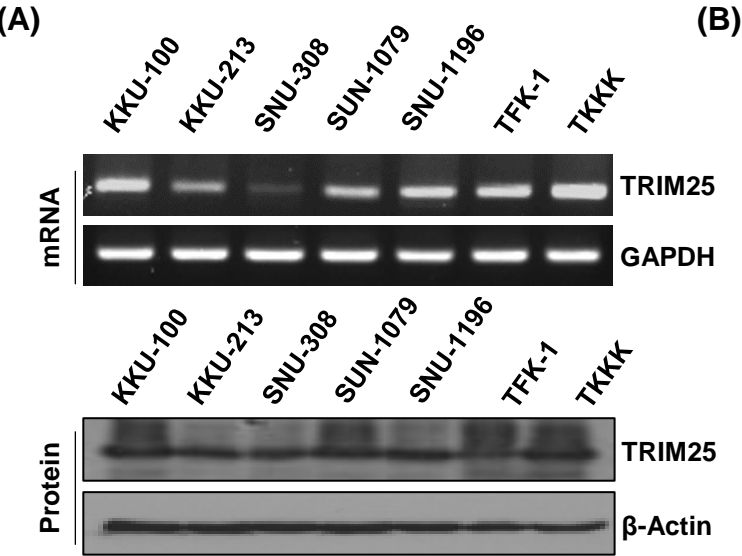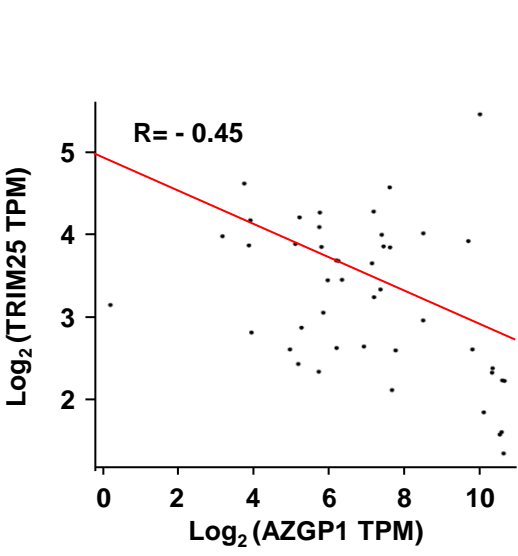

Supplement: Supplementary file 2 — Figure S2 [file JCMM-28-e18104-s003.pdf]

Supple Fig 3.

(A)

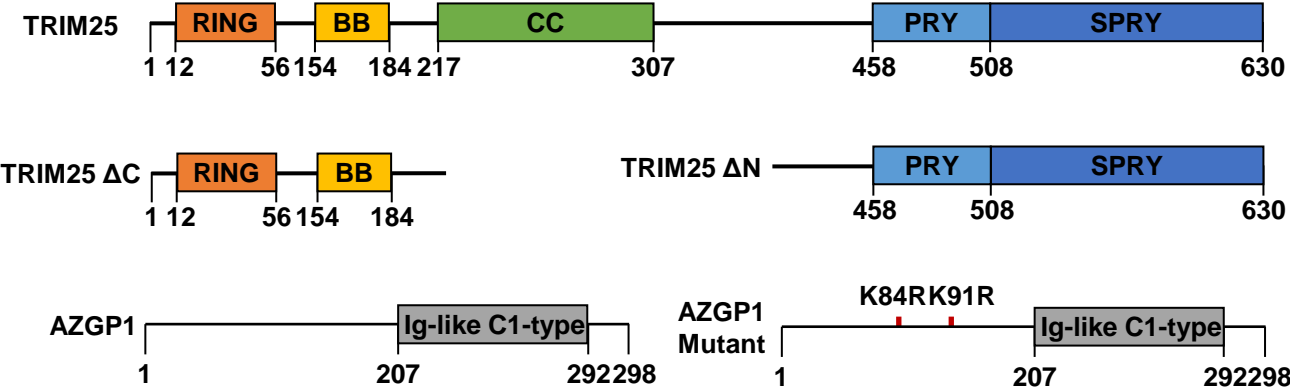

Supplement: Supplementary file 3 — Figure S3 [file JCMM-28-e18104-s001.pdf]
