# Supplementary material for: Degradation of AZGP1 suppresses apoptosis and facilitates cholangiocarcinoma tumorigenesis via TRIM25
Source: J Cell Mol Med. 2024 Jan 6;28(3):e18104. doi: 10.1111/jcmm.18104 (PMC10844717; doi:10.1111/jcmm.18104)
Supplement: Supplementary file 4 — Table S1 [file JCMM-28-e18104-s002.pdf]

Supple Table 1.

Genes		Sequence
AZGP1	Forward	5'-CAGAAGCAGCGGAGCATTCT-3'
	Reverse	5'-TGCCTCCCACTTCTGCTTGG-3'
TRIM25	Forward	5'-CAGACCTTGAAGGAGGAGATTG-3'
	Reverse	5'-GAGAGCAGTATGTGAACCTCTG-3'
GAPDH	Forward	5’-GGACTGAGGCTCCCACCTTT-3’
	Reverse	5’-CCTGCAGCGTACTCCCCACA-3’
